# Women’s perceptions of COVID-19 and their healthcare experiences: a qualitative thematic analysis of a national survey of pregnant women in the United Kingdom

**DOI:** 10.1186/s12884-020-03283-2

**Published:** 2020-10-07

**Authors:** Babu Karavadra, Andrea Stockl, Edward Prosser-Snelling, Paul Simpson, Edward Morris

**Affiliations:** Norfolk & Norwich University Hospital, Colney Lane, Norwich, NR47UY UK

**Keywords:** COVID-19, Corona virus, Pregnancy, Impact, Survey

## Abstract

**Background:**

The aim of this national survey was to explore pregnant women’s perceptions of COVID-19 and their healthcare experiences.

**Methods:**

Through patient and public involvement, a questionnaire was developed and advertised via the BBC website, Twitter and other online media during May 2020. The findings were analysed by qualitative thematic analysis. Women who are currently pregnant, or who have delivered during the COVID-19 pandemic were invited to partake in a national online survey.

**Results:**

One thousand four hundred fifty-one participants replied to the online questionnaire. Participants provided significant insight into the perceived barriers to seeking healthcare during this pandemic. These include ‘not wanting to bother anyone’, ‘lack of wider support from allied healthcare workers’ and the influence of the media. Other concerns included the use of virtual clinics antenatally and their acceptability to patients, the presence of birthing partners, and the way in which information is communicated about rapidly changing and evolving services. The influence of the media has also had a significant impact on the way women perceive hospital care in light of COVID-19 and for some, this has shaped whether they would seek help.

**Conclusions:**

This is the first ever reported study in the United Kingdom to explore pregnant women’s perceptions of COVID-19 and their subsequent healthcare experiences. It has also provided insight into perceived barriers into seeking care as well as maternal concerns antenatally, intrapartum and postpartum.

## Background

The impact of the COVID − 19 pandemic has been unimaginable and has resulted in a significant number of deaths throughout the world [1]. In the United Kingdom, hospitals have had to drastically change the way in which they deliver their services, and in many instances, suspend certain elements of care. During the COVID-19 pandemic, a national ‘lockdown’ was declared in the United Kingdom and as a result, all non-essential travel and contact with other individuals outside a person’s home environment was banned. Significant changes were also implanted in hospital and these included, social distancing measures, postponement of non-essential surgery and major changes in the way primary and secondary care services were delivered. In maternity care, many antenatal contacts occurred virtually, restrictions were imposed on birthing partner presence and clinicians has to use suitable personal protective equipment (PPE) as appropriate. At the time of writing this paper, the current number of deaths related to coronavirus in the United Kingdom is 46,706 (Gov.UK).

The changes in the delivery of maternity care in the United Kingdom and the impact of these changes on pregnant women and their birthing partners are not known. Maternity care is very unique, and it simply cannot stop due to the acute nature of the specialty. At present, no studies have been conducted to explore women’s views on the impact of COVID-19 on their pregnancy and medical and/or midwifery care. Therefore, the objective of our online study is to explore pregnant women’s’ perceptions of COVID-19 and their healthcare experiences. It is hoped that the findings will be used to ensure women are receiving holistic care that is tailored to their specific needs during this pandemic.

The Royal College of Obstetricians and Gynaecologists (RCOG) responded rapidly to develop an evolving guideline and information for women and their families on the impact of COVID-19 in pregnancy [2, 3]. The findings from the first study into outcomes of women affected by COVID-19 in the UK showed that women from a Black, Asian and Minority Ethnic (BAME) background were more likely to die from COVID-19 and also at higher risk of developing complications of COVID-19 [4]. This study is designed to explore pregnant women’s perceptions of COVID-19 and their healthcare experiences. It is designed to obtain insight into any barriers to healthcare during this pandemic and any concerns women have about any stage of their pregnancy. It is hoped that the voices of pregnant women from this study will be used to shape future maternity services.

## Methods

Ethical approval was obtained from the University of East Anglia (reference 2019/20–06).

### Design

A 26-item questionnaire (combination of open and closed-ended questions) was developed by a team of clinicians and academics through the guidance and support of patient and public involvement. Five women who are currently pregnant and five midwives of varying age, clinical background and ethnicity were asked on their feedback of the questionnaire with regards to the content and wording. The questionnaire aimed to explore pregnant women’s perceptions of COVID-19, the impact of it antenatally, intrapartum and post-partum through free text questions. It was also designed to explore any perceived barriers to care during this pandemic. Consensus of the content of the questionnaire and its design was determined as a group through discussion. The questionnaire is available as a supplementary file.

### Data collection

The questionnaire on Survey Monkey was advertised online between 1st May 2020- 9th May 2020 through the BBC website, Twitter and other online spaces facilitated by the research midwifery team. The local radio station also advertised the study. The data was collected over 9 days from Survey Monkey and once the response rate was above 1400, the questionnaire was deactivated as saturation was deemed to have been reached. The questionnaire was available to anyone who was currently pregnant or had delivered since the COVID-19 pandemic commended in the UK.

The data was collected and analysed daily and saturation was defined as no new themes being generated from the responses. We ensured that the questionnaire was advertised through many different online groups and through the BBC news website page to ensure we reached a wide range of participants.

### Analysis of data

The findings from the free text questions were analysed by qualitative thematic analysis [5–7] and the findings were contextualised to the clinical setting by all five authors on a Microsoft Excel spreadsheet. Some questions were analysed by simple percentages and presented in a table. The open-ended questions were analysed by thematic analysis. Thematic analysis is used to analyse opened-ended data (for example from a survey or interview) to identify and generate patterns from within it (called themes). This involved becoming familiar with the data set (reading the responses and understanding them), creating initial codes (attaching labels to different sections of the text), generating themes based on the respective codes (by grouping different codes together) and applying this to the context of our study question [7].

## Results

In total 1552 individuals opened the questionnaire, but 1451 participants replied and completed the questionnaire. One thousand two hundred twenty-one participants were prenatal and 230 were postpartum. Table 1 shows the different participant demographics.

**Table 1:** To show the participant demographic data

Demographic	Proportion, n (%)
**Age range**	
**18–24**	**37 (2.56)**
**25–34**	**964 (66.29)**
**35–44**	**447 (30.89)**
**45–54**	**3 (0.20)**
**Geographical location**	
**East of England**	**266 (18.33)**
**East Midlands**	**80 (5.54)**
**London**	**235 (16.27)**
**North East**	**42 (2.91)**
**North West**	**100 (6.93)**
**Northern Ireland**	**24 (1.66)**
**Scotland**	**75 (5.19)**
**South East**	**256 (17.73)**
**South West**	**148 (10.25)**
**Wales**	**56 (3.88)**
**West Midlands**	**84 (5.82)**
**Yorkshire And the Humber**	**85 (5.88)**
**Ethnicity**	
**White/White British**	**1334 (91.9)**
**Black/Black British**	**10 (0.68)**
**Asian/Asian British**	**50 (3.44)**
**Mixed race**	**33 (2.27)**
**Rather not say**	**4 (0.27)**
**Other**	**20 (1.37)**
**Highest level of education**	
**Less than secondary school qualifications**	**4 (0.28)**
**Secondary school qualification**	**163 (15)**
**Some university, but no degree**	**72 (4.98)**
**Foundation degree**	**43 (2.97)**
**Bachelor’s degree**	**621 (42.95)**
**Post-graduate degree**	**548 (37.9)**

Other important information obtained from participants showed that 2% were diagnosed with COVID-19, 6% had a family member diagnosed with COVID-19 in the same household and 58% were self-isolating due to government advice and personal anxieties about ‘catching the virus’.

### Identified themes

**Fig. 1:**
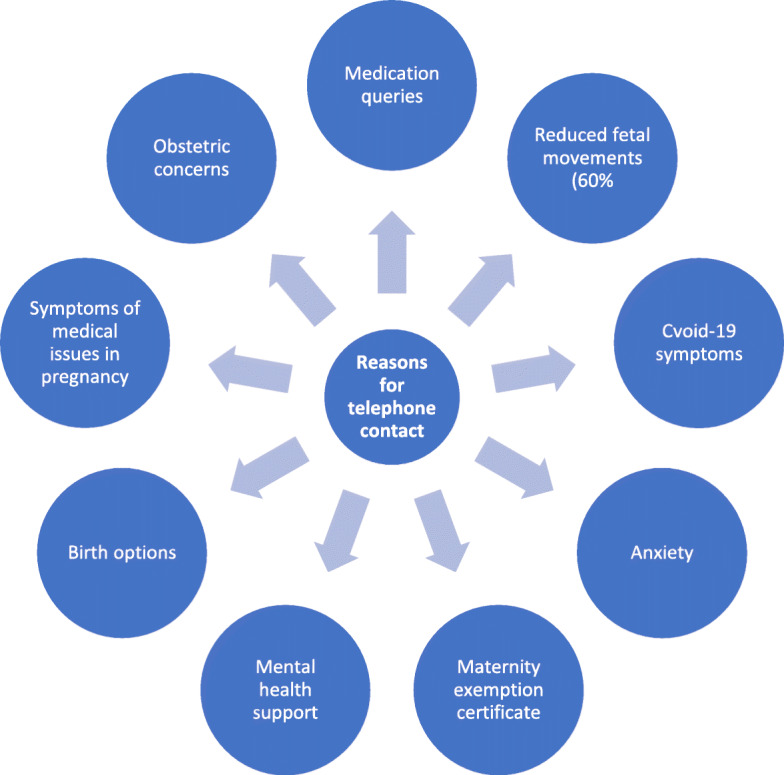
Chart to show the main reasons for contacting maternity triage during Covid-19

The main concerns and experiences discussed by participants were grouped under the categories of antenatal, intrapartum and postpartum care. Before discussing these themes, it is important to note why participants sought maternity care during COVID-19. Seventy-three percent of the cohort sought medical or midwifery help during COVID-19 lockdown. Figure 1 shows the main reasons as to why participants contacted maternity triage during COVID-19.

### Below is a summary of the concern’s participants expressed about their antenatal, intrapartum and postnatal care

#### Antenatal care

**Fig. 2:**
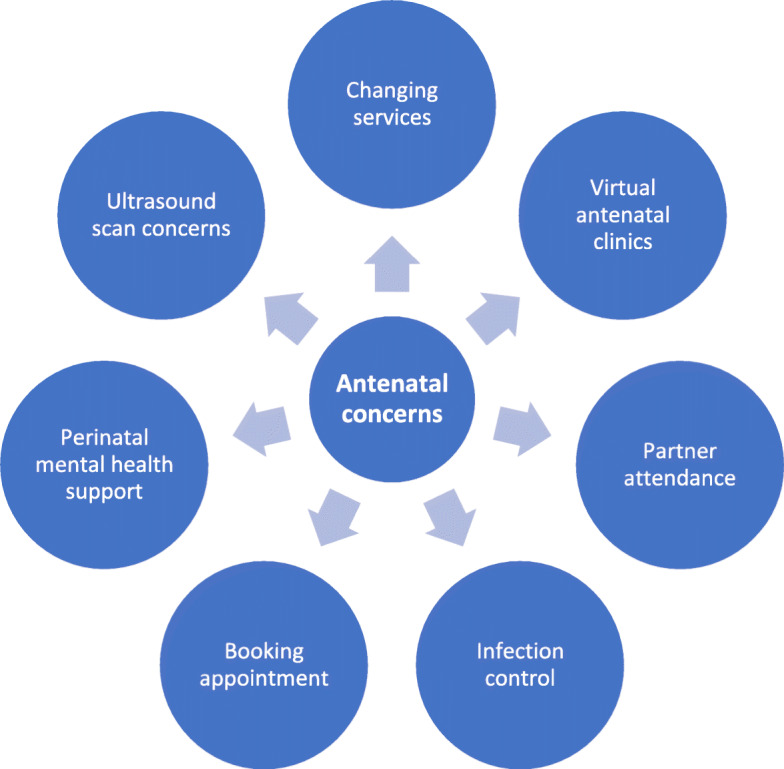
Chart to show the main antenatal concerns expressed by participants

Participants provided insight into the following concerns about their antenatal care (Fig. 2):

‘Virtual consultations’; whilst participants understood why these measures were in place, 62% felt that this provided ‘impersonal care’ and it affected how much information they disclosed to their healthcare worker. Two participants who had hearing impairments explained that they ‘could not communicate properly on the video link’ due to internet connectivity problems. Fourteen percent of participants explained that they were reluctant to speak about mental health issues over the phone. One participant explained that she did not want to disclose this information to her partner but isolating together made this difficult if asked about on the phone. Eleven percent of participants said that they felt ‘embarrassed to talk about mental health concerns over the phone’ and felt discussing sensitive issues over the telephone was not appropriate. Participants were worried that ‘things may not be picked up on the phone that they would normally in person’. Six percent of women had a previous stillbirth, and therefore would have preferred to have a face to face consultation to reduce anxiety. Twelve percent explained that they would have liked more written information about their care. Some participants resorted to the internet for further advice. A number of participants explained that their mental health related appointments were cancelled and felt that the level of support was significantly reduced.

A change in the way maternity services are accessed has meant women have had to travel to different locations for their care, and as such, public transport issues have made travelling a problem. Two percent of participants explained they were less mobile (due to wheelchair use) and therefore had problems accessing different departments in the hospital when attending for appointments. The different ‘zoned areas based on ‘COVID wards’ and ‘non-COVID wards’ made it difficult for these people to access the correct areas in the hospital and caused confusion. This was ‘made worse as partners were not allowed to attend’. Twenty-one percent of participants perceived that healthcare workers were ‘more concerned about COVID-19′ than pregnancy related issues itself.

Twenty-eight percent of women explained that they were having ‘regular scans’ for a maternal or fetal reason, however, due to COVID-19, the scan frequency had changed, and was often reduced; women wanted more information about how this could impact their pregnancy. Twelve percent of women ‘avoided the hospital at all costs’ and therefore did not attend for their routine scan appointments due to concerns about contracting COVID-19.

Some participants explained that their booking appointment did not feel ‘detailed’ and were concerned that some aspects of their care may have been missed. They were also concerned as the doctor did not have access to their full maternity records when conducting the appointment virtually.

The media played a significant part in women’s perception of hospitals and ‘risk of death’. Participants recalled news articles on those who had unfortunately died from COVID-19 and had concerns about either dying themselves or their baby. Fourteen percent explained that should they be diagnosed with the virus, then they were ‘worried about how healthcare workers will treat them’ and were concerned as to whether staff had the correct personal protective equipment. The use of hospital services and the risk of asymptomatic transmission from healthcare workers was also mentioned. Importantly, two participants from Afro-Caribbean backgrounds were told they were ‘more likely to die from COVID-19’ but they were both unsure as to why.

Thirty-two percent explained they were worried about the impact of the virus on medical co-morbidities such as asthma and ‘not knowing where to go if symptoms of asthma worsened’. Twelve percent provided insight into the ‘conflict’ they experienced within themselves if asthma symptoms were worsening and ‘whether to get help or not’.

Over 60% of women sought help for reduced fetal movements. They explained they felt reluctant in contacting the hospital should they be asked to attend for fetal monitoring. One woman described this as a ‘double edged sword’ where she wanted to seek help for reduced fetal movements, but yet, felt concerned about COVID-19 exposure in hospital.

Some women with asthma raised concerns about its symptoms being similar to COVID-19 and so wanted more information on when to come to hospital should symptoms worsen. Medical reasons for contact also included pre-eclampsia, gestational diabetes, urinary tract infection, vaginal bleeding, pelvic pain, mastitis, chickenpox exposure and ruptured membranes.

Twelve percent felt that their questions were ‘irrelevant’ in the current COVID-19 pandemic and therefore felt ‘embarrassed’ to seek support. Some women explained that they perceived the **‘**threshold for seeking help had gone up during COVID-19’ and felt reluctant to seek further help. Eighteen percent of women said that they thought their concerns were not ‘serious enough’ and thought that healthcare workers were only dealing with ‘the seriously unwell’ patients or those with COVID-19.

#### Intrapartum care

**Fig. 3:**
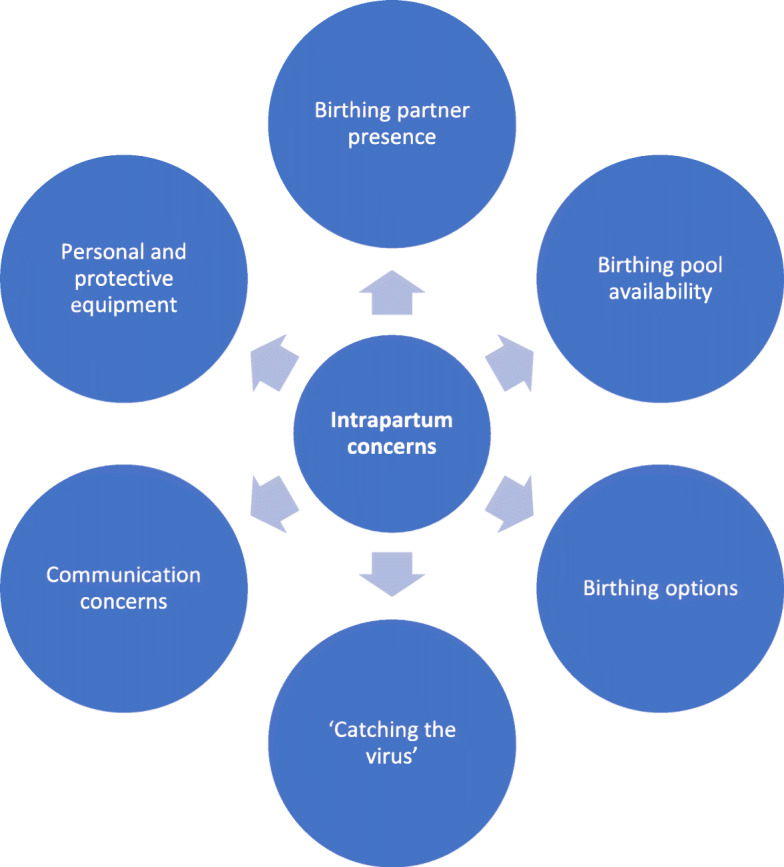
Chart to show the main intrapartum concerns expressed by participants

Participants provided insight into the following concerns about their antenatal care (Fig. 3):

Partner presence was the most common theme discussed for intrapartum care. Participants explained that they were worried about partners not being allowed to be present at all during labour or during active labour only. They were also worried about ‘being alone’ should there be an intrapartum problem.

The risk of ‘catching COVID-19’ was not surprisingly, a common theme discussed. Participants were concerned about ‘transmitting COVID-19 to the baby’ due to recent news articles and online press.

Some participants expressed concerns about not being able to ‘have a normal delivery’ and the ‘risk of a C-Section’ if diagnosed positive for COVID-19. Eleven percent discussed their concerns about ‘being separated from the baby at delivery’ if COVID-19 was confirmed, and ‘how they would be cared for by staff’ They also expressed concerns related to the ‘stigma of being diagnosed’ and whether staff would feel comfortable looking after them.

The majority of participants highlighted concerns about the availability of a birthing pool and any restrictions on its use. Eleven percent of women felt they were ‘denied’ the opportunity to deliver in a birthing pool due to COVID-19. In addition, concerns were raised as to whether induction of labour would be cancelled or significantly delayed. Participants were worried about staying on the ward in a bay with other patients who may be asymptomatic of COVID-19. A number of women were concerned about a hospital delivery and therefore enquired about homebirth options.

Forty-two percent of women said that they did not want to seek support as they were ‘worried about being invited into hospital for a review’ and the risk of coronavirus transmission. These concerns stemmed from the governments advice to ‘Protect the NHS’, media reports on the lack of personal and protective equipment (PPE) for healthcare workers, the risk of contracting COVID-19, fear of attending alone and waiting in a bay or room with other patients. Participants explained that whilst they were happy to attend the hospital if required as an emergency or routine care, they felt unclear about any specific precautions that they should take prior to attending; ‘should I be wearing a face mask? Do I buy my own face mask? Or will I be given one upon arrival to the hospital?’

Two participants with a hearing impairment were concerned about staff communicating with them during labour with a protective mask on.

#### Postnatal care

Breastfeeding support was the most common theme discussed as participants were concerned about how these services will operate postnatally. Participants also expressed significant concern about the reduced midwife and health visitor contacts in the community. Some participants explained thy were worried about postnatal depression and were unsure where to seek support from.

Women explained that social isolation based on government advice resulted in either a complete lack of support from family and friends, or a significant reduction in support. A number of participants explained the struggles they have faced post-delivery and the lack of support from family members. The lack of wider support from physiotherapy and allied health professionals in the community has meant women have had to ‘find other ways of coping’ 12% explained the challenges they faced when seeking support from the General Practioner in terms of access to prescriptions and face to face appointments. Three percent of participants mentioned feeling ‘confused by what support the pharmacist is now able to offer. Two percent of participants were ‘unable to access dental care’ in terms of emergency treatment or routine care.

## Discussion

### Main findings

The COVID-19 pandemic has been an unusual situation and its impact on expectant and new mothers has been significant. One thousand four hundred fifty-one participants replied to this questionnaire. The findings from this study provide significant insight into women’s perceptions of COVID-19 and their healthcare experiences. There is a significant amount of information on the concern’s women have during their antenatal, intrapartum and postnatal period. Whilst the study has yielded many findings, the main areas of concern focussed around coronavirus transmission, the use of virtual clinics antenatally and their acceptability to patients, the presence of a birthing partner, and the way in which information is communicated about rapidly changing and evolving services. Fifty-nine percent of participants felt there were barriers to accessing healthcare for pregnant women during COVID-19 lockdown and discussed a multitude of reasons. These included the use of virtual consultations, reluctance to discuss mental health issues virtually, the perceived threshold for seeking help, the risk of coronavirus transmission, the role of the media and changes in the way services are delivered. The media appear to have shaped some participants perceptions about hospitals and their risk of ‘catching Corona’; subsequently, this has had an impact on health seeking behaviour as some participants felt ‘too worried’ to obtain medical or midwifery help. Those participants who lived in a rural location or coastal location were also less likely to seek help due to barriers in physically accessing healthcare.

Whilst the number of participants from BAME backgrounds was only 6%, the information they provided in terms of barriers to health in particular was striking. They explained the perceived ‘stigma’ they experienced when seeking medical or midwifery experience. For one reason or another, this group of participants detailed negative experiences with healthcare workers and felt they were ‘not taken seriously’. The use of PPE by healthcare workers resulted in some participants experiencing ‘communication barriers’; this was particularly discussed by those with hearing impairments.

### Strengths and limitations

This is the first ever reported study in the United Kingdom to explore pregnant women’s perceptions of COVID-19 and their subsequent healthcare experiences. A significant number of changes have occurred in maternity care since the pandemic, and this study has shed light onto what women perceive their care to be like at this time. It has also provided insight into perceived barriers into seeking care as well as maternal concerns antenatally, intrapartum and postpartum.

As this study was conducted online and as a survey, researchers were not able to ask further questions to clarify the responses. Whilst this was a national survey, the vast majority of responses were from participants who were white British and therefore, the results may have been different if more individuals from Black, Asian and Ethnic minority (BAME) groups replied. Whilst it is difficult to quantify the exact number of participants who may have been able to respond to the questionnaire, it is important to note that there were 640,370 live births in England and Wales in 2019 [8]. The survey largely reached those who are engaged in current affairs and with online access; therefore, resulting in responder bias. However, this work has enabled us to develop a qualitative study involving semi-structured telephone interviews with pregnant participants from BAME backgrounds specifically to explore the impact of COVID-19 in greater detail. This is in progress currently.

### Implications of findings

Our findings show that the impact of COVID-19 has been significant to participants using maternity services. Participants with co-existing mental health problems also appear to be feeling stigmatised through the lack of support services available. Participants explained in great detail the impact of not being allowed to bring a birthing partner to the hospital or place of care had on them. There does not appear to be strategies in place to support patients in the antenatal, intrapartum or postnatal period.

Participants discussed their reluctance to address mental health concerns with healthcare workers during the pandemic. Some participants discussed the challenges of discussing mood related concerns over the telephone. There is emerging evidence to suggest that pregnant women are experiencing significant anxiety during the pandemic [9–11]. Further local and national strategies should be considered by policy makers to help further support this group of women.

It is clear that women’s’ healthcare is affected during any global healthcare emergency; with the most recent to COVID-19 being Ebola and H1N1 infection [12, 13]. Maternity units across the United Kingdom have had to significantly change the way they deliver their services to patients in view of this pandemic. There appears to be stark differences between different units in the way care is delivered, and as expected, this is causing anxiety amongst patients. Participants provided insight into the use of social media and Facebook forums to compare the type of care they have received.

Whilst maternity units have had to react acutely to this pandemic, the findings from this study do raise questions as to the way services can be delivered into the future. There may well be more of a focus on virtual clinics through the use of other communication methods. However, this study provides strong evidence that research into the safety and efficacy of these techniques. It also highlights that it will be important to have high levels of patient and public engagement when such service changes are being considered. A recent national survey of junior doctors in the United Kingdom has also explored this from a healthcare worker perspective [14].

The latest UKOSS study [4] of more than 400 pregnant women hospitalised with COVID-19 found that 55% of pregnant women hospitalised were from a BAME background. There is also increasing published evidence, that pregnant women who are from a BAME background may experience health inequalities during pregnancy for a number of reasons and this needs to be explored further [4]. Our study, that involved 6% of participants from a BAME background (who are particularly more likely to be affected with an adverse outcome in pregnancy or the puerperium) shows they experienced some form of challenge in seeking medical or midwifery attention in pregnancy. Our study has provided the basis for further research with participants from a BAME background in the form of semi-structured interviews individually or via focus groups.

## Conclusion

In summary, the COVID-19 pandemic has had a significant impact in the way healthcare is currently being delivered in the UK. Maternity services have undergone a significant change. Whilst these changes may be temporary, they highlight significant questions as to how services should be delivered in the future. It cannot be assumed that the current way of service delivery is necessarily acceptable to patients, and it is important to involve the voices of women and their families when designing such services. We hope the findings from this study will be used to shape future maternity services include the use of virtual antenatal care.

## Data Availability

The datasets used and/or analysed during the current study are available from the corresponding author on reasonable request.

## Abbreviation

BAME: Black, Asian Minority Ethnic
